# Software-In-Loop Simulation of an Underwater Wireless Sensor Network for Monitoring Seawater Quality: Parameter Selection and Performance Validation

**DOI:** 10.3390/s21030966

**Published:** 2021-02-01

**Authors:** Alberto Clavijo-Rodriguez, Victor Alonso-Eugenio, Santiago Zazo, Ivan Perez-Alvarez

**Affiliations:** 1 Information Processing and Telecommunications Center, Universidad Politécnica de Madrid (UPM), Av Complutense 30, 28040 Madrid, Spain; santiago@gaps.ssr.upm.es; 2 Instituto para el Desarrollo Tecnológico y la Innovación en Comunicaciones (IDeTIC), Universidad de Las Palmas de Gran Canaria (ULPGC), 35017 Las Palmas, Spain; valonso@idetic.eu (V.A.-E.); ivan.perez@ulpgc.es (I.P.-A.)

**Keywords:** underwater communications, environment simulator, sensor simulator, electromagnetic underwater wireless sensor network, software-in-loop simulation

## Abstract

In this work, a real-time software-in-loop simulation technique was employed to test and analyse an underwater wireless sensor network. This simulation should facilitate the deployment of the real network and helps guarantee the network’s expected behaviour. We study duplicated packets, one-way delay, and power consumption to analyse the network’s leading parameters. Evaluating production-ready software in simulated conditions eases effective deployment. This method will ultimately allow us to establish these parameters, test the software before the deployment, and have an excellent understanding of the network’s behaviour.

## 1. Introduction

Underwater wireless sensor networks (UWSN) are useful communication infrastructures for seismology, oceanography, marine life monitoring and surveillance, natural disaster prevention and control, integrity checks of oil and gas facilities, and military tactical operations [1,2,3,4,5].

These applications’ network requirements differ considerably in terms of bandwidth, maximum acceptable latency, redundancy protection, and environment deployment, among other parameters. Some of these applications may require quasi-real-time monitoring, control systems [6], or early warning systems [7]. These networks typically use mesh topologies where node devices are interconnected (partially or fully connected). Therefore, node behaviour must have been validated prior-deployment, because nodes are critical for the performance of the network as a whole. The validation process should contemplate the three main functions of nodes operation: data acquisition, data storage, and data communication. Nodes should offer a well-known input interface to accept incoming data from sensors and probes. They should also be capable of storing a significant amount of data and avoiding data loss in case of hardware or network failures. Finally, nodes must respond faster within time-critical applications, and adapt to node disconnections in multi-hop network architectures. As an example, in [8], communication between a central station and nearby autonomous robots was performed using a multi-hop network topology. The system required the optimal design of transport protocols to enable robust transmissions. On the other hand, low power consumption must be taken into account when designing these node devices, thereby reducing maintenance costs related to energy-storage (such as trips to recharge batteries or solar panel-enabled buoys). Hence, energy efficiency is an essential issue in UWSN, as stated in [4,9].

These highly restrictive network requirements, along with the costs associated with deploying the infrastructure underwater, justify the use of simulation and emulation tools to verify the expected behaviour of these critical node devices.

In this work, a software-in-loop (SIL) simulation is proposed to evaluate production-ready firmware of device nodes. This simulator executes the software that will be finally embedded within each node with only minor modifications. Therefore, the cost associated with software development is minimised. In other words, the use of this proposed SIL scheme validates already-developed software without the need for framework-specific implementations or adaptations to generic simulation tools [10]. Other popular tools, such as DESERT, SUNSET, and Aqua-Net, need substantial modifications to their code in order to be used live.

In this study, we evaluate the real-time operation of an electromagnetic underwater sensor network (EM-UWSN) based on the STANAG 5066 communications stack. These nodes provide a particular application with the communications infrastructure—in this case, the monitoring of a coastal sewage emissary. Monitoring this emissary is an issue that is currently under study and will be addressed, as it is a necessary step before the network’s deployment. This will be the objective of a forthcoming paper where we will be able to compare the results obtained in our simulation model.

This application simulates the effectiveness of adaptive network topologies because seawater outfall includes a diffusion section that eases sewage dilution (among others). Furthermore, marine outfalls have a static infrastructure, an ideal scenario for deploying real-time seawater quality monitoring. However, the results obtained in this study and the proposed method and technology could be extrapolated to many other similar applications, such as oil spill detection in fuel transportation facilities.

This scenario was chosen concerning the economical, social, and environmental impacts on society. The water quality of touristic cities is the main issue, and local authorities expend incredible effort toward guaranteeing healthy coastal environments for tourists and local people. Furthermore, nowadays most European countries cannot manage the increasing amount of urban wastewater, as stated in [11], and outfalls operate beyond their capabilities, so there is a real need for monitoring marine outfall emissions which, in the best cases, discharge sewage far from the coastline (2 km in the case of study), and are only slightly diluted, using multi-port diffusion in order to improve sea absorption.

**Figure 1:**
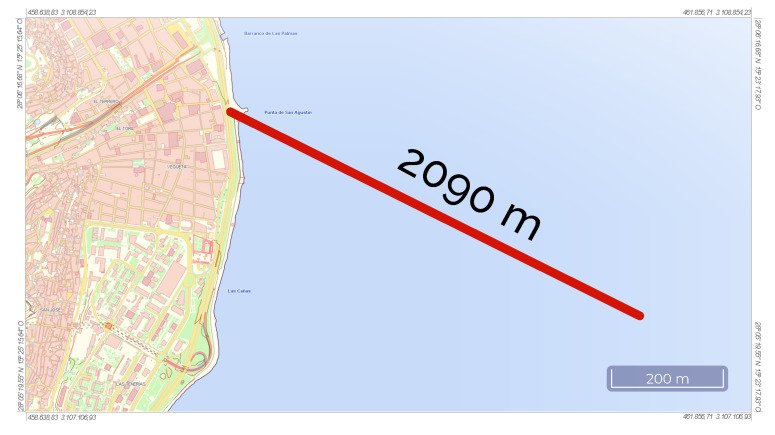
Marine outfall location.

The selected scenario is a marine wastewater outfall near Las Palmas de Gran Canaria, located in Gran Canaria, a particular island of the Canary Islands. Figure 1 shows the location on a map.

For this scenario, a setup of an UWSN with 34 virtual nodes was tested by using two different simulation settings. All simulations were set with the same base scenario configuration, but two different settings were studied. In the first setting, the marine outfall operated in normal conditions. In contrast, the second case corresponds to a critical situation where the outfall is leaking just before the expected diffusion section. In this situation, the nodes located near the breakage must send high priority alarm packets, operating in an early warning mode. These two settings allow the network’s performance to be tested in soft and harsh conditions.

To select proper parameters, report undesired network behaviours, anticipate possible solutions, measure network performance, and determine which nodes are more critical are decisive steps to design real deployment with affordable cost.

This paper is structured as follows. Section 2 introduces the previous achievements on this line of research. Section 3 provides a complete analysis of the state-of-the-art solutions regarding UWSN simulation. Section 4 submits the simulation scenario and the proposed network topology. The simulator architecture considered in this paper is presented in Section 5. The application developed in this work is presented in-depth in Section 6. The sensor mock-value generator software is presented in Section 7. The methodology used to set the network parameters and run the simulations is detailed in Section 8. The experimental results are presented in Section 9. Finally, conclusions are extracted in Section 10.

## 2. Previous Achievements

This research presents part of the results obtained in the national research project Harnessing Electromagnetic Underwater Communication Networks by Self Configurable Deployments (HERAKLES). This project aims to develop new strategies for underwater node configuration and deployment and characterise and analyse the channel for electromagnetic communication links.

Previous work in our research has been to take in situ measures to model and characterise the underwater radio channel in shallow waters [12], in a previous project named UNDERWORLD. Several antennas were designed and tested in low-frequency bands. The agreement between attenuation measurements and simulations at different distances was investigated, which made it possible to validate the simulation assemblies and design different system communication layers.

The results of this investigation led to the fit of a parametric path loss model, allowing for narrow-band communications while keeping a reduced amount of noise [13]. Moreover, it was established that the existing communication framework could be used for self-localisation techniques by means of specifically tailored algorithms.

Later, and belonging to the current project, the environment was simulated with the above-mentioned SIL technique [10]. This technique made it possible to emulate the operation of an EM-UWSN in real-time, using a STANAG 5066 stack as a basis that would subsequently be part of the development of a real underwater sensor node. The STANAG 5066 (STANdard AGreement 5066) is widely used in military communication, particularly for HF protocols, often being used in scenarios in which throughput and reliability are low. Therefore, STANAG 5066 serves as an optimal choice for its use in UWSN applications for electromagnetic underwater radio frequency communication (EM-URFC).

One of the goals of this project was to research the way EM signals are transmitted underwater, not only in theory but though experiments conducted in a real environment as well. The project also features a multiple input node interface for offshore networking (MINION) as an integral part of an UWSN. A software-in-loop platform is used to emulate the real-time operation of a STANAG 5066 stack that replicates the behaviour of an underwater network of MINIONs.

## 3. Related Work

The dispersion of wastewater diffusion from coastal ocean sewage outfalls [14,15,16,17,18] has been extensively studied. These studies took in situ measurements in different ways and combined the collected data to characterise the diffusion models and create three-dimensional maps of the effluent plume distribution from the sewage outfall. The measurement methods included autonomous underwater vehicles (AUV) and combinations of these vehicles with divers. Ramos et al. [18,19] used an AUV to study the shape and estimate the initial dilution of a sewage outfall plume. Bonin et al. [20,21] combined divers and a lightweight AUV to analyse the effect of a marine outfall and its toxic waste on the surrounding submarine ecosystems. Understanding how the dilution of sewage outfall plume works is a useful resource to design a system capable of monitoring its near field.

None of the above research used sensor networks for long-term and continuous space-time monitoring. To address this, Adamo et al. proposed a wireless sensor network for water quality monitoring based on surface buoy probes [22]. Nevertheless, monitoring anchorage areas for large ships and the harbour’s entrance channel could be problematic, especially when measures are taken on the sea surface. Furthermore, sensors located on the sea surface detect the far-field outfall; it would make more sense to monitor the influences of many sources. The study of near-field discharges requires the sensors to be close to the seabed. In this way, early warnings are triggered by the source of the problem. If the non-diffusion path of the pipe breaks, leaks could be detected when the failure happens.

The seabed sensor networks have also been studied. King et al. [23] studied the acoustic channel for a seabed network in which the nodes were close to the bottom. Person et al. [24] addressed a long term seabed observatory of geo-hazard areas. Both Chandrasekhar et al. [25] and Tan et al. [26] addressed the problem of location in underwater sensor networks where nodes are in the seabed.

Although the aforementioned works use sensor networks on the seabed, none of them work for real-time monitoring. Moreover, all of them use acoustic waves as the transmission channel, leading to high propagation delays and low data rates. These problems, along with their dependence on environmental channel behaviour, are not easily circumvented, since they are intrinsic to acoustic communication [27]. However, electromagnetic (EM) communication has advantages over acoustic and optical communication in shallow water coastal environments [28]. Specifically, magnetic induction (MI) is not affected by long propagation delays, multipath propagation fading [29]. The lack of a significant signal propagation delay and the predictability of channel response, together with a high bandwidth range that is wide enough to allow for quiet, potentially concealed transmission, make MI a promising candidate for underwater communications [27].

The costs and efforts related to testing and deploying new systems make it useful to develop a complex simulation environment [30]. There are different simulator environments to validate protocols and algorithms [31] in wired and wireless networks [32]. This software has been mainly developed for terrestrial networks, such as NS-2 [33] or OPNET. There are extensions that complement the base functionality of these tools. As acoustic communications are the commonly accepted method for underwater environments, based on NS-2, Aqua-Sim [34] can effectively simulate acoustic signal attenuation and packet collisions in underwater sensor networks, supporting three-dimensional deployment. Aqua-Sim NG (next generation) [35] is based on NS-3. MIRACLE [36] is an extension for NS-2 that allows multiple protocols within each layer and facilitates the design of cross-layer algorithms by defining a dedicated communication bus. There are tools that work over NS-2/MIRACLE, such as SUNSET [37,38] and DESERT [39]. These tools integrate real hardware into the simulation process to emulate a more accurate environment. In the frameworks mentioned above, users must implement a network protocol specific to the simulation environment, and once they want to test it on a real experiment, they must re-implement the protocol again, this time to make sure it works on the real system. Hence, SUNSET and DESERT propose hardware-in-loop simulations combining real hardware with traditional simulations to reduce protocol development into a single effort [40].

In contrast, simulator-based protocol stacks Aqua-Net [41], and its next-generation SeaLinx [42], are OS-based stacks because they use pure Operating System APIs, and run in real-time; each protocol runs as a single process. Additionally, UnetStack [43] is based on an agent framework, which supports both real-time and discrete event simulation modes. Zhu et al. [44] compared these tools and evaluated their performances.

There are so many of these tools that there are articles trying to help researchers find the right tools [31,44,45,46,47,48]. However, most of these tools require the developer to extend the simulator’s codebase by implementing the system to be evaluated. The fact that the simulation code is different from the production code means that their results are less reliable.

Some of these tools state that the code is reusable from simulation to production. Issac et al. [49] compared some of these tools. However, this only works in that order and a protocol tested and compiled previously cannot be used without having to re-write the code. In other words, this option is not compatible when the communication stack has been previously implemented and compiled. Other researchers are trying to establish a new paradigm in underwater MAC protocol development, where simulation and deployment use the same code [50,51]. However, once again, the developer needs to adapt the protocol code to the framework API.

As an alternative, it makes sense to produce a simulation environment that tests production-ready code at simulation time. The reliability of the node software is critical. Thus, the system application requirements can be set, tested, and validated using the SIL simulation technique. It removes issues of model validity, as the same software is reused in the design, coding, and testing phase [52]. This new environment provides substantial knowledge of network behaviour at an early stage of the project, helping one to make better decisions in the whole process.

The information about the emissary chosen for this study was extracted from [53], an official document of Canary Island Government. Additionally, Aguirre et al. presented a detailed explanation about its history and repairs [11]. It outlines the multi-port diffusion section’s installation during its last repair, which relays a simulation of the discharged fluid’s dispersion.

## 4. Scenario Definition and Network Design

**Figure 2:**
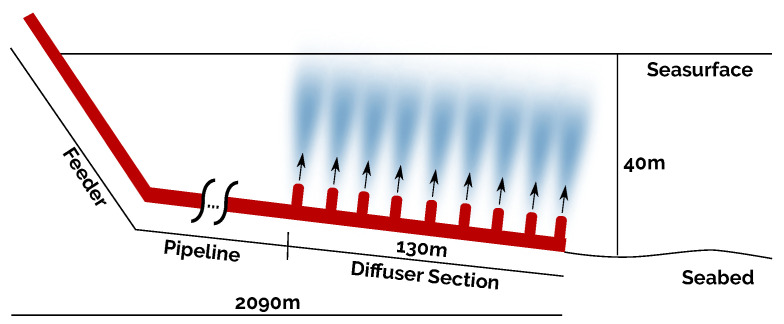
Marine outfall schema.

The marine outfall structure has three different sections: the feeder, the pipeline, and the diffuser section, as shown in Figure 2. The discharge pipe is approximately 2 km long. Its diameter varies from Ø1 m at the beginning (the connection with the feeder) up to Ø0.4 m at the end (the diffuser section). The diffuser section consists of a multi-port diffuser where equidistant jets release the sewage into the sea. It is submerged 40 m below sea level and has an extension of 130 m.

The proposed sensor network is deployed around this discharge pipe. Nodes will be connected between them through electromagnetic links. The network is deployed in shallow waters, so acoustic and optical alternatives have effects that are challenging to manage. Acoustic waves are affected by the multi-path effect, and the optical links are not an option because of turbidity. Furthermore, electromagnetic communications have a power consumption of less than the acoustic one, which is a crucial reason for this kind of application.

Two main issues constrain the nodes’ distribution: sensors must measure the water quality in the pipeline’s immediate vicinity while guaranteeing the electromagnetic link’s optimal performance and robustness. According to Hunt et al. [17], a high concentration of residual substances starts to decrease around 13 m from the seabed. Therefore, the maximum distance between nodes and pipe has been set to 5 m as a design criterion. In order to guarantee connectivity between nodes, the distance between two neighbour nodes is restricted to a maximum of 15 m, in accordance with the measurements and simulations previously performed and described in [10,12,13].

**Figure 3:**
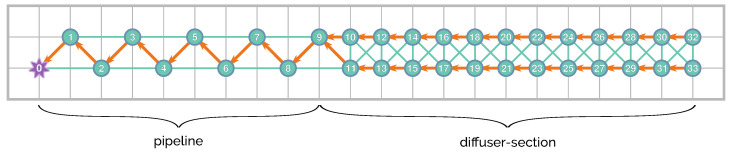
Node distribution—the background grid is 10 m per division.

The proposed network topology follows two different patterns, whether it is located in the pipeline or the diffuser sections. It consists of a different number of nodes according to the necessary spatial resolution of monitoring, as shown in Figure 3. This figure illustrates the 34-node distribution over a 10 m square grid. The resolution requirements at the diffuser section are greater than along the pipeline. An alteration in the measured magnitudes is expected to appear closer to the diffusion section, while the outfall operates in regular conditions. However, it is necessary to monitor the pipeline to prevent possible leaks and to warn accordingly.

The network follows a zigzag pattern with 10 nodes around the pipeline, from the feeder up to the diffuser section, where a 2 × 12 node mesh is deployed. The zigzag pattern detects leakages along the entire length of the pipe while having a low amount of nodes per unit of distance (compared with the setup around the diffuser section); this pattern also detects leakages on both sides of the pipe, regardless of the direction of the tide.

Additionally, there is a master node that serves as a gateway with the outside world. It is the first one of the network and would be responsible for sending inter-network packets and receiving all the internal messages from all the nodes (acting as a sink node).

In this study, the number of nodes within the zigzag pattern has been limited to 10, covering only 100 m of the pipeline. However, in a real scenario where the central station is land-based, the number of nodes could increase up to the shoreline, or use long-range transmission systems attached to the master node.

## 5. Simulation Architecture

The proposed SIL testing technique helps to run, test, and validate production-ready source code within a modelled environment.

The simulation system relays on three differentiated blocks or modules: the node block, which includes the software that will be used in a real scenario; the sensor simulator block that generates sensor measurement samples, reading them from a file (refer to Section 7), and then sends to nodes encapsulating raw data using the actual communication protocol that is used by real sensors (DS18B20 sensor [54] is used for temperature readings, and the same packet frame format is used for salinity readings); and, finally, the environment simulator block, which provides the simulated network interface by simulating the communications among all network nodes.

**Figure 4:**
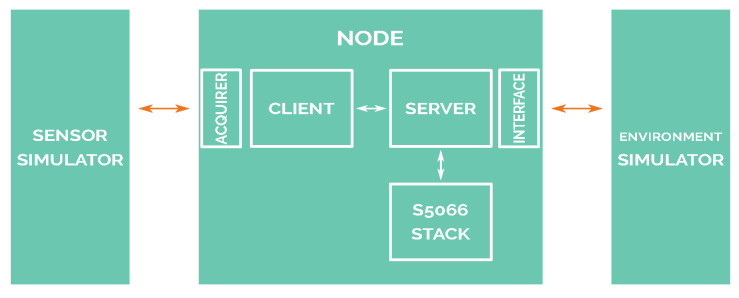
Software-in-loop diagram.

The relations and blocks of this architecture are shown in Figure 4. These blocks are completely isolated from each other. Furthermore, communication among all of the connected blocks of the diagram is based on TCP/IP.

### 5.1. Node

A client–server architecture has been deployed, following the architecture proposed by Alonso-Eugenio et al. [10]. The original software architecture involved in the underwater node is made up of three main executable software programs: client, server, and STANAG 5066 stack. These processes communicate between each other using TCP/IP sockets.

The client software block is where the application defined in Section 6 runs, reading values from the sensor present for every node. It also handles the communication with the STANAG 5066 stack, implementing the SIS layer communication protocol. The server software coordinates the communication between STANAG 5066, the client, and interface block. This block knows when a packet from STANAG 5066 is addressed to be processed by the client or must be sent to the interface (and thus, sent to the simulator). The client, server, and STANAG 5066 blocks, and their interrelationship and functionality, are described in deep detail in [10].

There are only two parts of the code that differ from the production-ready software: the sensor acquirer and the interface block. These two parts are responsible for communicating for the node with both the sensors and the network. The code differences related to the acquirer block rely only on the software interface used to communicate with the sensor simulator. The sensor simulator will emulate the exact packet frames used by a DS18B20 temperature sensor (there will not be any changes in the packet decoding procedure). However, instead of using a serial interface, the sensor simulator uses TCP/IP interface, so modifications are minimal.

The interface block needs to be changed in the same way as described by Alonso-Eugenio et al. [10]. The interface block would communicate with the real modem in a real scenario, but in this case, the interface block has to implement the communication protocol used to communicate with the environment simulator.

The simulator defines three different states of node operation: transmitting, receiving, and idle. The energy consumption related to each one of these states has a different unitary cost. Accordingly, the consumption of each node depends on the amount of time it spends at each state [55], and it can be computed using Equation (1).

(1) $$E_{comm}=T_{tx}\cdot e_{tx}+T_{rx}\cdot e_{rx}+T_{i}\cdot e_{i}$$

where $T_{tx}$, $T_{rx}$, and $T_{i}$ correspond to the time the communication interface spends transmitting, receiving, and in the idle state, respectively; and the terms $e_{tx}$, $e_{rx}$, and $e_{i}$ are the unit costs of the states. The values of these parameters, $e_{tx}$, $e_{rx}$, and $e_{i}$, are set in Table 1. The unit cost of each of these states takes into account the energy consumption of every subsystem active during the period in which the node is in every state.

### 5.2. Environment Simulator

The environment simulator establishes a virtual environment and generates all the physical and communication parameters of the link between nodes. In this environment, each node has its corresponding location coordinates to evaluate communication channel effects, such as signal to noise ratio (SNR), and its associated bit error rate (BER), among others.

In this study, we assume that the network is already established. In terms of the routing default path shown in Figure 3, the nodes would first need to route their messages to the node with the shortest path to the master node. In this figure, the arrows show the default path. On the other hand, the lines represent other possible route paths. It is important to point out that the distance between adjacent nodes still allows a stable link.

These other possible route paths are used by the node in case of a node failure, or if a node acknowledges disconnection or a communication interruption with its default node. To do so, this network proposal has mechanisms for adapting the node’s routing table using auto-discovering algorithms. However, these algorithms are not in the scope of this work, and the alternative routing tables have been pre-established.

The network topology in the vicinity of the pipeline has a high dependency in each link of the chain; therefore, these alternative paths guarantee network connectivity if a node is down.

This environment simulator has been developed, tested, and documented in a previous, related study [10], which modelled the electromagnetic link under seawater conditions, and characterised the architecture used in this study. Moreover, the STANAG 5066 standard for EM-UWSN has been already tested and validated using a SIL simulation environment.

Furthermore, an environment simulator records the communication transactions between nodes. This strategy facilitates the analysis of the results obtained. For example, the consumption of each node can be indirectly computed by using the power consumption related to the transmissions used, as mentioned in the section on nodes.

### 5.3. Sensor Simulator

Temperature and conductivity sensors were selected as the two parameters to monitor seawater around the marine outfall because they are related to water quality [56] and are usually included within multi-parametric sensors.

As physical sensors will not be present during simulation, a sensor simulator was designed and implemented in order to simulate all of the network sensors and to keep all of their data synchronised. The physical sensors that would be used in a real deployment are DS18B20 for the temperature sensor, and we assume another sensor with the same packet definition of DS18B20 for water conductivity, which can be used to compute salinity.

In that way, a sensor simulator generates the readings of the sensors in the same packet format as the selected digital sensors would do. As input, a sensor simulator reads a file containing all the values of one sensor. This file has been created following the procedure shown in Section 7. Every datum in the file must contain a mark relative to the beginning of the simulation, so it is possible for the simulator to synchronise the readings of all of the sensors present in the simulation.

**Figure 5:**
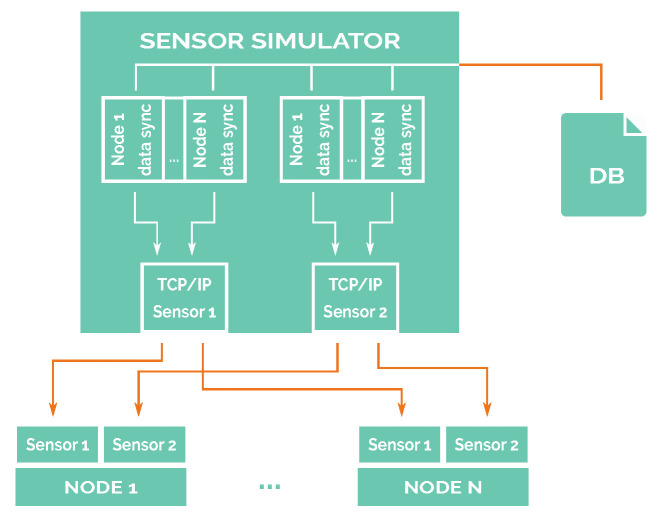
Sensor simulator diagram.

After reading the values from the corresponding files, the simulator will conform a new packet, following the packet format defined by the DS18B20 integrated circuit, and then, it will send that conformed packet through TCP/IP to the corresponding node, as shown in Figure 5. From the point of view of the node, there will be minimal differences between reading sensor data from a TCP/IP socket and reading them from a serial port. Under GNU/Linux, the procedure for reading data from a socket and a serial port is exactly the same, so the only change is where the data is expected to come from.

## 6. Application

In this section, we detail the functional logic implemented by each node to operate as a sensing device in a wastewater outfall monitoring network. This application has been developed with the purpose of demonstrating the feasibility of a practical water quality monitoring application, and it is focused on evaluating the simulation platform and environment under these requirements.

In terms of communication, the network must transmit the read values obtained by sensors from each node towards the master node in a reasonable time. In this way, each node acts as a relaying device, not only sending its own data but redirecting data from further nodes to the node which is ahead in the route to the master.

As mentioned before, there are two different settings simulated in this work. In the first setting, the wastewater outfall is operating in normal conditions; thus, the nodes are storing and sending data regularly every flush time. The second setting simulates some leakage along the pipeline; then, an alarm mechanism should be triggered. When a node reads data which lies outside a previously defined range of values (defined in Section 6.2), the data will be tagged with a warning flag, and this packet is then relayed with high priority to the master node, following the node’s operation routines detailed in Section 6.4.

The algorithm used to detect anomalies and mark the warning data packets is detailed in Section 6.3.

### 6.1. Network Settings

There are some parameters that can be configured to adjust the network’s behaviour. All parameters are set as the node execution parameter. These parameters have their own addresses, and relay the node’s address and flush time.

As it was already presented in Figure 3, there are several hops between the master node and the farthest node from it, so it was necessary to define a strategy where nodes collect data from sensors until they transmit them to the relay node. This strategy implies defining the time between transmissions of each node and the difference in these times between two neighbour nodes.

The flush time is defined as the instant (referred to the minute of the current time) in which a node will send to its configured relay node all the data it has on its buffer, even if there are data that have been read by its own sensors or data that were received from a neighbour node and it has to act as a relay. Moreover, the difference between the flush times of two neighbour nodes is defined as propagation time and it is an indirect configurable parameter which is a function of the flush time.

The other key parameter is relay node. It refers to the node address where every node will send all their data so that data flow will follow the relay node configuration.

The values of these parameters have significant impacts on the network’s performance. The setup of these values will be discussed in Section 8.

### 6.2. Node Settings

The application-specific configurable parameters of each node are its sample period, its temporal resolution, and the range of regular outfall activity operation for each measured parameters.

Sample period is the time elapsed between two consecutive sensor measurements. In this particular scenario all the sensors are set to a fixed value.

The temporal resolution of the node’s activity depends on the sample period and the importance of the acquired sensor data. For example, if several measurements contain the same relative value and it is within the expected normal values range, it is not strictly necessary to send it repeatedly; thus the energy efficiency of the network can be improved. On the other hand, when a huge irregularity or a sudden variation happens within consecutive measurements, it would be essential to notify the master node to act in consequence. In this case, the temporal resolution matches the sample period.

### 6.3. Warning Labelling Procedure

As mentioned previously, the normal values are considered to be a range in which a selected magnitude is expected (for this application). The expected normal values must be specified as execution parameters for all of the nodes.

When the acquired sensor values lie outside of this range, it is considered an anomaly. In this situation, the data packet generated is labelled with a warning flag.

### 6.4. Node’s Operation

**Figure 6:**
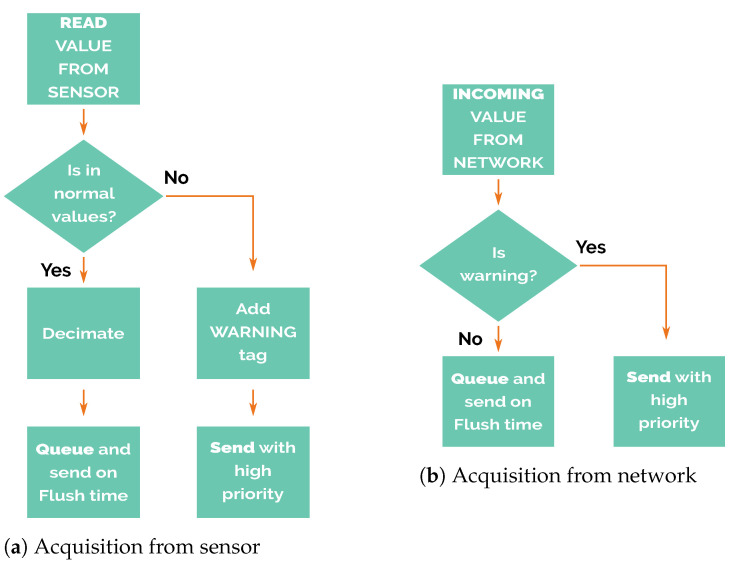
Acquisition Phase.

The node’s operation could be divided into two different phases. The first one corresponds to the reception of new incoming data, whether they are acquired from its own sensors or received from packets transmitted from other nodes (Figure 6). When new data are acquired by the sensors, the warning labelling procedure mentioned above checks if the value is within the range of expected values. Otherwise, it proceeds with the decimation of values. The decimation process discards twenty-nine out of every thirty samples, resulting in 1 sample every 30 min. The reduction in queued samples reduces the resolution of the monitored magnitude. For this reason, decimation only is applied to values inside the normal values range. The adaptive resolution allows maximising the efficiency of the future packet transmissions. In the event of values being above the expected values, it generates a new data packet and labels it with a warning flag that must be sent as soon as possible.

After the acquisition phase are the preparation and transmission of data packets. It is essential to highlight that in this phase the node would operate completely differently depending on the warning flag status.

**Figure 7:**
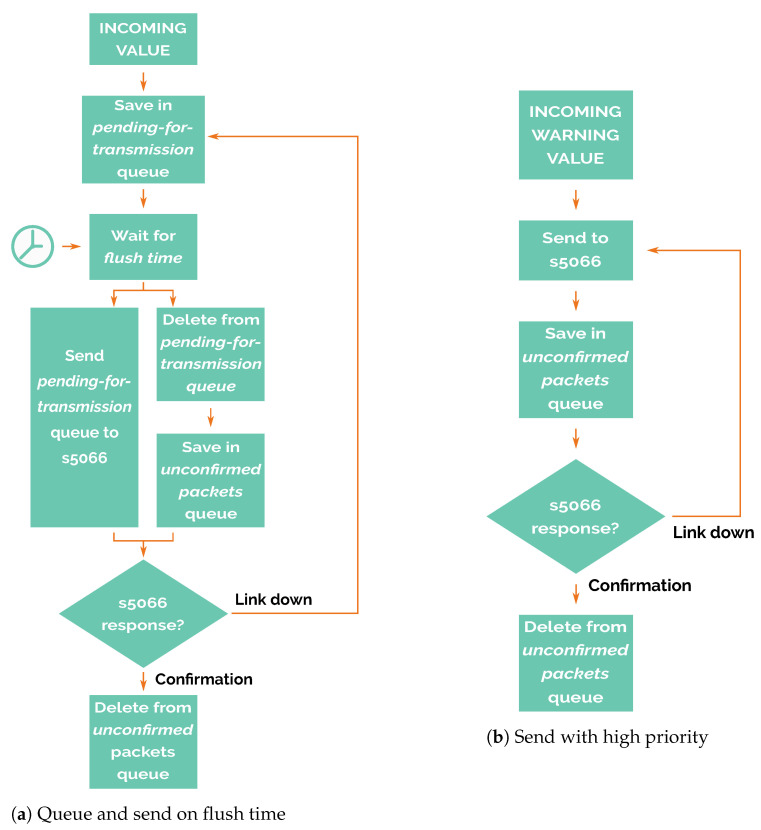
Preparation and transmission phase.

If the warning flag is not set, then the newly acquired data are saved for later transmission, when the flush time begins (detailed in Figure 7a. In other words, it stores the read values obtained from its own sensors, or the packets received from other nodes in a pending-for-transmission queue.

When the flush time event occurs, packets are popped from the top of this queue and are sent to the STANAG 5066 to be transmitted. These packets are then stored temporarily in an unconfirmed packets queue until STANAG 5066 confirms that the packet has been successfully sent. In case of communication failure, packets stored in the unconfirmed packets queue are moved back to the pending-for-transmission queue to be sent in the next flush time. In this way, the node tries to avoid packet losses, an important feature of this system.

The packets sent to STANAG 5066 cannot be temporarily deleted and then reinserted after STANAG 5066 has rejected that packet, because STANAG 5066 does not guarantee that the notification of the rejection includes the complete payload of the original message, so all of the content has to be kept in the unconfirmed packets queue.

It is possible that messages which have been sent to STANAG 5066 and stored in the unconfirmed packets queue are actually sent to other nodes and received by them, but the confirmation message that deletes the message from unconfirmed packets queue is lost. In this case, these messages will be resent again, duplicating the values received. This situation is intentional because it is preferable to avoid packet losses at the expense of receiving duplicated packets.

On the other hand, if the warning flag is set (Figure 7b), packets are never stored in the pending-for-transmission queue and only are stored in the unconfirmed packets queue. This feature gives high priority to alarm packets, so nodes try to send these messages regardless of flush time.

Alarm packets have a temporal resolution higher than the regular ones because no decimation process reduces the available bandwidth; they use it all. Therefore, the time a warning packet takes to arrive at the master node only depends on the number of transmissions over the electromagnetic link between nodes in the routing chain without waiting for flush time. This feature has been considered to find a system able to switch between energy-efficient mode during the regular operation and high-accuracy mode when the early warning system is triggered.

### 6.5. Packet Structure

**Figure 8:**
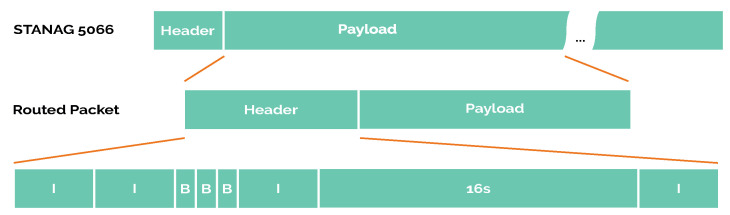
Packet structure.

According to STANAG 5066, the packet format is a 16 byte fixed-size header and a variable-size payload (maximum 100 bytes), as shown in Figure 8. This payload is built by our node software from the routed package format, made up of a fixed-size header and a variable-size payload.

- I: Origin address;
- I: Destination address;
- B: Remaining hops to discard the package;
- B: Amount of hops since the packet was sent by the origin;
- B: Packet flags where the alert flag is included;
- I: Epoch (in seconds) of the source node when creating the message;
- 16s: MD5sum of topology from the previous node;
- I: Payload size,

Where *B* is an unsigned char of 1 byte, *I* an unsigned integer of 4 byte and *16s* a 16 byte string. These make a 35 byte fixed-size header.

The payload of sensor readings always has the same format:

(2) payload={id:message_id,sensor_id:reading_value}

where *message_id* is an integer as a unique message identifier, *sensor_id* a char that defines the kind of sensor, and *reading_value* a float number. This payload has 55 bytes. The sum of the header and payload reaches 90 bytes, so it is below the STANAG 5066 payload limit. Therefore, the size of each STANAG 5066 packet is 106 bytes.

## 7. Sensor Reading Generator

The sensor simulator requires a dataset with magnitude values and times in which each sensor performs a measure, as described in Section 5.3. A sensor reading generator software has been developed in MATLAB to accomplish this task, which generates the files that the nodes will read as real sensor measurements.

This software creates a dataset from a signal that may include the following factors:

- Mean values and basic temporal variation
- Spatial variation of magnitudes produced by the diffusion section of marine outfall.
- Limited time incident of a punctual focus emission within the pipeline.
- Noise.

Both space and time variations of the magnitudes are defined within datasets created by the sensor reading generator, meaning that we do not need to define a differential diffusion equation that characterises the emission, but we can define the effect on the values measured by sensors instead. Furthermore, the datasets generated for this study are approximations of the data published in [57] from an oceanographic station close to the selected scenario but not at the specific location. Before any real deployment is carried out, a more accurate model is needed to guarantee that the simulation settings agree with real values. Additionally, the range set for identifying normal or emergency values must be re-set for each node.

### 7.1. Mean Values and Elemental Temporal Variation

Values have been adapted from real data collected and made public by the Oceanic Platform of the Canary Islands (PLOCAN), who serve data collected by oceanographic stations [57].

**Figure 9:**
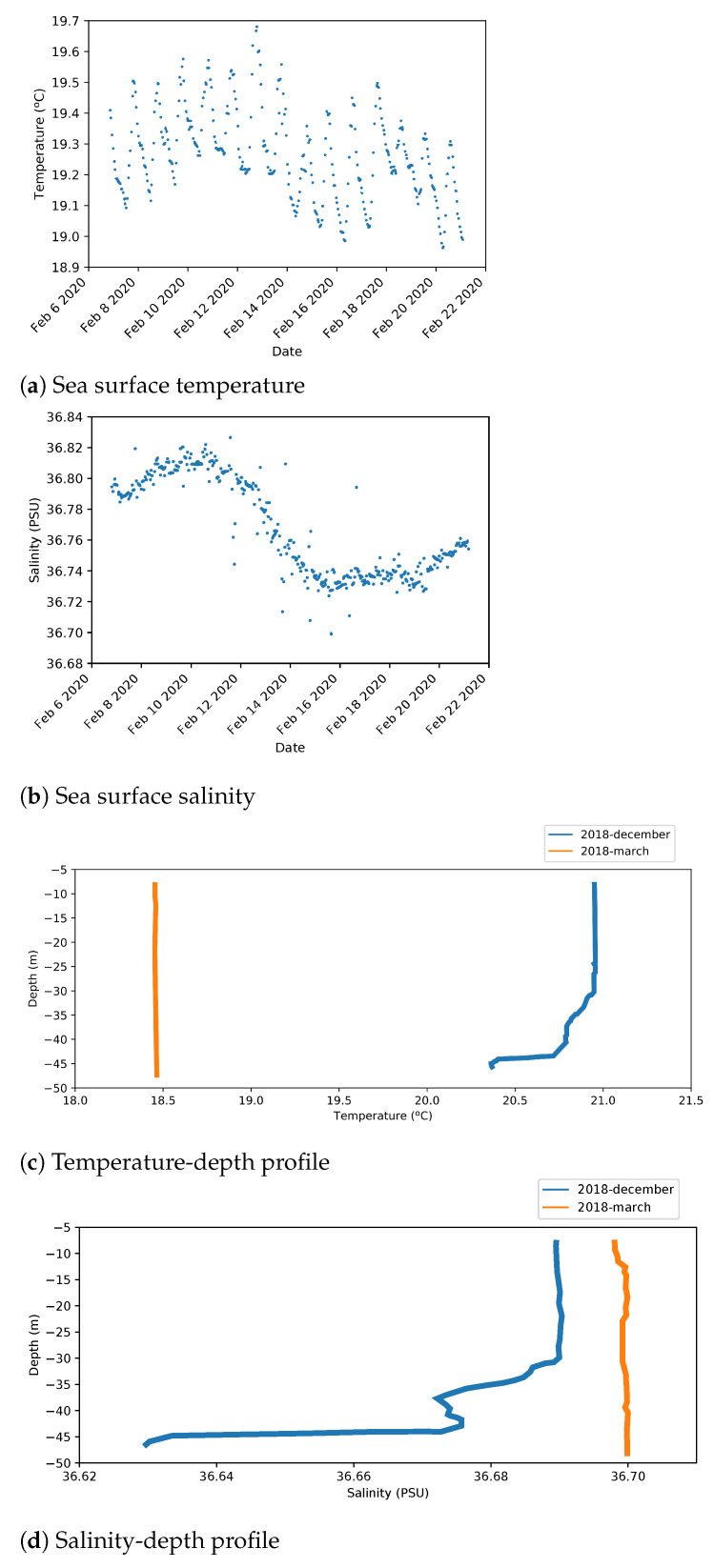
Real measures from the Platform of the Canary Islands (PLOCAN).

Figure 9a,b shows instantaneous temperature and salinity values measured at sea surface from an oceanographic station close to the outfall area.

Figure 9c,d shows the variation of these magnitudes according to depth.

From this information, the following values have been extracted at a depth of 40 m:

- Mean water temperature is around 18.5 ${}^{\circ }$C.
- Temperature values include an oscillation about 0.2 ${}^{\circ }$C because of day/night variation.
- Mean salinity value is 36.7 PSU.
- Salinity has a 0.1 PSU variation with a long period of around two weeks.

Time variation of magnitudes has been simplified by sinusoidal functions described by Equation (3), which was defined taking into account the shape of Figure 9a,b:

(3) $$V=\bar{V}+\Delta V\cdot sin\left(2\pi f\cdot t\right)$$

where $\bar{V}$ is the main value offset, ΔV is the amplitude of its oscillation amplitude, and *f* is the frequency of the oscillation.

### 7.2. Outfall Influence across Space

Around the multi-port diffuser outfall, seawater and discharged fluid mix to produce variations in the regular temperature and salinity. The salinity of the discharged fluid is less than the seawater’s, and this change is detected as a variation in electrical conductivity. The salinity values were computed using the polynomials developed by Millero et al. [58].

Fluids discharged by the multi-port diffuser produce a variation in seawater’s temperature and conductivity, which is measured by the nodes. The variation is greater for nodes within the diffuser section but varies smoothly as the nodes move away through the pipeline. A logistic function approximates the slight variation, whose transition matches the beginning of the multi-port diffuser. This means that nodes far from the diffuser section are not affected by the outfall influence; nodes close to the transition between pipeline and diffuser section receive it with an intermediate value; and nodes within diffuser section receive it with the maximum value. The maximum value results in a temperature rise around 1 ${}^{\circ }$C and a salinity decrease of around 20 PSU.

### 7.3. Punctual Focus Emission Incident

The incident consists of a punctual focus emission in the middle of the pipeline. The sensor data generator algorithm lets us add an extra offset that will mean that magnitudes get away from the normal values range scheduled for some nodes. Nodes close to the leak must detect the anomalies and generate warning messages with a high priority of transmission, such as an early warning system. This feature guarantees these nodes will operate in harsh conditions, allowing us to analyse the network’s performance for transmitting messages tagged as warnings. The leakage location is set in a middle point on the pipeline between nodes 5 and 6, 46 m from the beginning of the multi-port diffuser. We assume that the effluent plume will modify the measurements of nodes 4, 5, 6, and 7 with different intensity, node 5 and node 6 being the only ones which should generate alarm packets. The effects of this incident are set to last 12 h, with a 1 h rising edge and 2 h falling edge.

### 7.4. Settings of Experimentation

The datasets used for the proposed settings are made up of the aforementioned factors. The settings to simulate soft conditions include mean values and basic temporal variation, spatial variation of magnitudes produced by the diffusion section of marine outfall and noise contributions.

**Figure 10:**
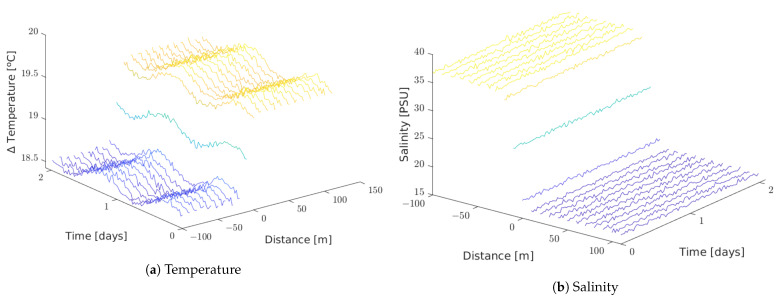
Soft setting.

Figure 10 shows temperature and salinity values read by each node in the soft simulated setting.

The dataset for the simulation of harsh conditions includes a time-limited incident of a punctual focus emission in the pipeline and the ones mentioned previously.

**Figure 11:**
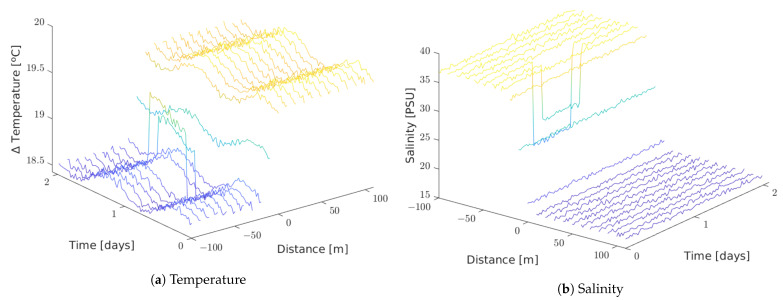
Harsh setting.

Figure 11 shows temperature and salinity values read by each node in the harsh simulated setting.

## 8. Methodology

Once the feasibility of the SIL approach for real-time simulation of the STANAG 5066 protocol stack in underwater RF has been demonstrated and validated, as Alonso-Eugenio et al. stated in [10], we must choose a specific application to carry out an integration test of the system.

The node’s operation workflow was developed and described in Section 6, to provide the necessary operating logic for the sewage outfall monitoring system. This study evaluates these procedures as proof that the proposed SIL architecture is the right tool to evaluate the whole system before its real deployment.

On the one hand, the packets sent by each node should be received by the master node, but as explained in Section 6, the node workflow prevents any packet from going missing. Even so, the possibility of receiving some packet more than once is known.

On the other hand, the early warning system feature was evaluated. This test requires a comparison between soft and harsh conditions, so two settings have been simulated. The first one consisted of the regular operation of the marine wastewater outfall where sewage goes down the pipeline, and discharges in regular conditions through the multi-port diffuser. In the second setting, the outfall leaks before the expected diffusion section, so in a known time interval, a discharge occurs in the middle of the pipeline. Nodes close to the leak should generate alarm packets which spread quickly through the network towards the master node whilst the leak is active, and once the leak is fixed the system should be back to regular operations. The delay time of regular and warning packets was compared by analysing the packets’ one-way delay.

The different settings we simulated consisted of two different datasets, which were selected in the sensor simulator module before running the simulation. Each dataset included the time and measurement value of each sensor, as is described in Section 7.

By using these two different settings, this work focused on producing some indicative results that can validate the feasibility of this kind of simulated network. The replicability of the simulations depends on two main pillars: values read by sensors and the settings of nodes during deployment. The first factor depends on the configuration of the sensor reading generator described in Section 7. The network settings are presented below.

### 8.1. Scheduled Parameters

When the network is deployed, each node must be set with a specific configuration to fulfil its role within the network. In this Section, parameters explained in Section 6.1 and Section 6.2 acquire values.

The sample period of sensor readings has been set to a value per minute. The temporal resolution of alarm packets matches with the sample period because it is the highest resolution of the system, while the temporal resolution of the regular ones has been set to one value every thirty minutes. This means that only one out of thirty reads is stored, and the rest are discarded.

The packets spread through the network because of consecutive flush events of every node in their paths to the master node. Thus, in order to avoid collisions, the flush times of neighbouring nodes should not be set to the same value. This difference between the flush times of neighbouring nodes, the propagation time, will determine the delay time that packets will take to arrive at the sink node.

Data transmitted by any node in the network will take the propagation time per node on the routing chain to arrive at the master node. Besides, there is an initial delay between the instant the value is read by the sensor, and the scheduled flush time of this node.

Equation (4) describes an estimated delay (normalised by the number of hops made by the packet) of the data sent by every node.

(4) $$t_{{}^{delay}{\;/\;}_{hop}}=\frac{t_{propagation}\cdot N_{hops}+\Delta t_{to\_flush}}{N_{hops}}$$

where $t_{propagation}$ is the value assigned to the scheduled propagation time parameter, $N_{hops}$ the number of hops between a node and the master node, and $\Delta t_{to\_flush}$ the time spent waiting for the scheduled flush time.

If propagation time is set with a low value, nodes could trigger their own flush events while continuing to receive packets from their neighbours. Furthermore, the linear network topology implies that nodes close to the master node work with a higher data volume than those farthest away. A propagation time that is too high could cause a node’s buffer to overflow. The configuration of the flush time should prevent a network overload. The network topology of this study, in addition, means a single node is responsible for merging two branches, so this node must receive each branch at different times.

The propagation time has been set to 15 min for the whole network. In order for the node that merges the branches to prevent collisions of packets coming from both branches, the nodes within the mesh topology flush their collected data every hour, with a 30 min delay between branches, and nodes within zigzag topology flush theirs twice an hour. This way, the merge node can send the data that came from a branch before it receives data from the other one. This schedule is set based on a calculation that determines the maximum transmission time (at this data rate) for the accumulated data coming from further nodes, to be around 10 min.

Temperature and salinity range of values under normal conditions are known. This information is used to set the normal values for sensors on nodes in the pipeline. This detects fissures or leakage in the pipe path. However, the expected range of values in the diffusion section is different due to the fluids that emerged from the multi-port diffuser, so the normal values of sensors on the mesh topology should contemplate this variation. Warnings generated in these cases will signal when the emission is lower or higher than the expected. These parameters have been defined as temperature normal values (TNV) and salinity normal values (SNV).

Parameters in Table 2 follow the specifications described above to achieve the expected network behaviour. Each node is set with its own configuration, and when the simulation is over, the analysis scripts are run to calculate the averages and get the results, as in the next section.

### 8.2. Simulation Parameters and Procedure

The complete network of 34 nodes has been simulated 10 times for each setting, with the same scheduled parameters. The analysis of the simulation is focused on evaluating the implemented logic within the application layer by studying duplicated packets, one-way delay, and power consumption.

The duplicated packets parameter tries to analyse how reliable the network is with regard to packet reception. We calculate the duplicated packets by comparing the packet ID that each node has sent to and received from the master node. Duplicated packets are calculated for each node by averaging the duplicated packets this node sent, for all of the simulations.

Almes et al.’s definition [59] has been adapted, in which one-way delay is the time difference of time between the source that sent the first bit of a packet to the destination at wire-time, and the destination that received the last bit of that packet. In our case, wire-time has been replaced by the time in which a packet is sent to STANAG 5066. The time difference between when a packet is sent by a node to STANAG 5066, and when the master node application receives this packet from its STANAG 5066 determines the one-way delay. This time is divided by the number of hops to normalise the delays of packets that come from each node. As a result of the duplicated packets received, the amount is not constant in all simulations, so an average makes no sense. Rather, one-way delay study analyses the aggregate packets in all simulations.

The study of the power consumption is computed by the time every node spends in each of the three possible operation states (transmitting, receiving or idle). As described in Equation (1), each of the times will be multiplied by corresponding unitary cost, resulting in the total energy spent by each node. The unitary cost of every operation state has been measured in laboratory, as detailed in Table 1, and corresponds with power consumption in mWh/s.

The dataset made up of values from sensors has up to 48 h of valid readings. After all of the values present on the dataset were read, the simulation was run for an extra 5 h to transmit all of the remaining buffered packets.

## 9. Results and Discussion

Running all real-time SIL simulations defined took seven weeks to complete. We ran twenty iterations for the two settings, following the methodology presented in Section 8. The packet’s traffic analysis gives information about the duplicated packets and delays on transmissions, and the associated power consumption was studied. In the next subsections, the functionality of the node workflow is analysed using the aforementioned parameters. However, this analysis focuses on the system’s capacity to facilitate this knowledge. Hence, the quality of the application is secondary to the capacity to analyse.

As mentioned in Section 6.4, due to the specific algorithm design, no packets were lost during the simulations. This analysis allowed us to determine that the designed algorithm was correctly coded and implemented.

### 9.1. Duplicated Packets

The study of the duplicated packets was based on comparing the logs generated by the source and destination nodes, so it gave information about what node generated packets were received once, twice, or more times. However, it lacks information about where each duplication was produced.

The packet management algorithm described in Section 6 completely removed the packet loss at the expense of creating duplicate packets.

**Figure 12:**
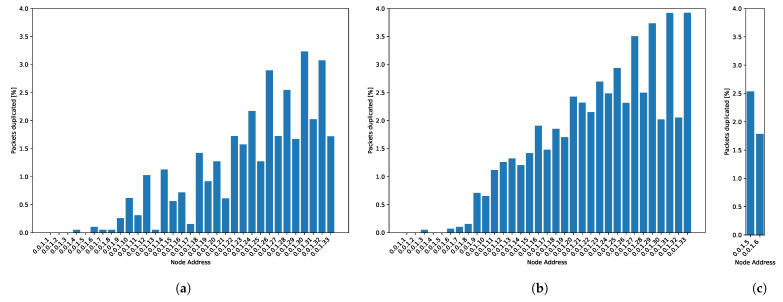
Duplicate packets: (**a**) Simulation without incident. (**b**) Regular messages for the harsh conditions setting. (**c**) Alarm packets for the harsh conditions setting.

Figure 12a shows the average of duplicated packets for the soft conditions setting. The probability of duplicated packets increases for the furthest nodes. This result is in accordance with the fact that duplication in closer nodes will also cause duplication of the buffered packets coming from nodes further away.

Figure 12 shows duplicate packets for the setting with harsh conditions. When harsh conditions produce alarm packets, duplicate packets grow by around 2%. The resolution of alarm packets is thirty times higher than the regular messages, and the amount of alarm packets is around eight times higher than the regular ones because of the duration of the punctual focus incident, described in Section 7.3. Alarm packets are not stored in queues waiting for flush times; they are directly sent to STANAG 5066 because of their priority status. We cannot appreciate a high impact on duplicated messages in alarm messages.

### 9.2. One-Way Delay

As mentioned previously, the difference between the transmission and reception time of a packet calculates the one-way delay. This parameter can be considered as one of the main parameters to evaluate the early warning system’s performance.

**Figure 13:**
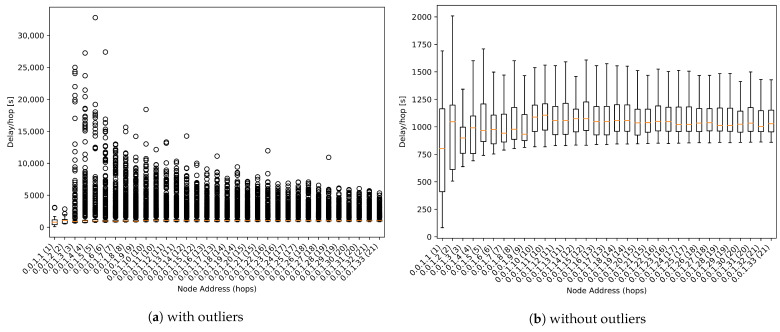
One-way delay boxplot.

The result shown in Figure 13b is the aggregate of the packets from all simulations with harsh conditions. In Figure 13b outlier values have been removed to facilitate analysis.

As we can observe in Figure 13b, the median value is around 1000 s per hop for all nodes. This value is close to the expected 900 s, which is the value set as propagation time for regular packets (see Table 1).

We can also appreciate that there are plenty of outliers with values up to 20 times higher than the median value. As its evaluation is not being addressed by this work, these results can be used for future works to detect some parameters to be improved.

**Figure 14:**
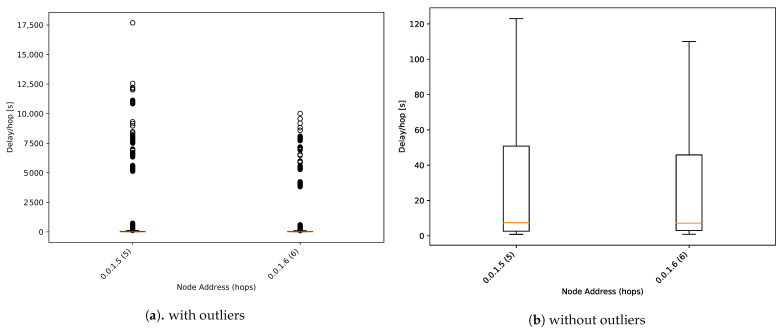
One-way delay boxplot for alarm packets.

Figure 14 shows a one-way delay boxplot for alarm packets. The median one-way delay of involved nodes has been reduced from 1000 s to 10 s per hop. Regular messages need to wait for the scheduled flush time, whereas alarm packets are transmitted directly. This feature gets the alarm packet to arrive at the master node quickly, generating an early warning system with a high-resolution of 1 min between samples. This feature increases the power consumption analysed in the following section.

However, some outlier values are very far from the median value. In those cases, they reach around 4 h per hop, unacceptable for a real deployment. In the context of this study, this is an example of the proposed system’s capacity for detecting unexpected behaviour of how the network manages the alarm packets.

### 9.3. Power Consumption

**Figure 15:**
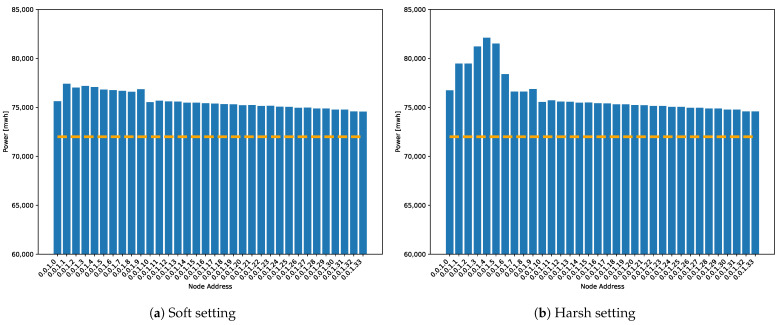
Power consumption per node. The dashed line marks the base consumption related to the idle state that each node consumes during the simulation time independently of its activity due to the application of this experiment. For clarity, the minimum value shown is 60,000 mWh.

Figure 15 shows the average power consumption of nodes in both simulation settings: with soft and harsh conditions.

During soft simulations, power consumption of the mesh topology increases linearly by branches because the number of packets increases with each link, and transmission time increases similarly. For nodes within the linear topology, the power consumption is higher than the nodes within the mesh topology because there are double the number of flush events. Furthermore, during harsh simulations, transmitting alarm packets boost the power consumption of the nodes that generate alert packets and the consumption of nodes that transmit those packets towards the master node through the network. As it was explained in Equation (1), power consumption depends on the time each node spends in each state. Transmitting alert packets considerably increases the time the node is in the transmitting state. This growth can be attributed to sending alarm packets.

## 10. Conclusions

This article is one further step towards a real implementation of underwater wireless sensor communications focused on a real application, a marine outfall in Gran Canaria Island. We are currently looking for extra funding to build the underwater nodes and contacting local authorities to achieve real deployment. This will be the objective of a forthcoming paper where we will be able to compare the results obtained in our simulation model.

Over an ad hoc simulation architecture, validated in previous works [10], we developed an application layer, and ran a complete integration test. The used STANAG 5066 stack is part of a real-world implementation of an EM-based underwater node, within the Spanish Government’s project HERAKLES. The main objective of this work was to validate the complete network behaviour and performance before the real deployment.

The proposed software-in-loop validation approach tested the production-ready code of the nodes with minor modifications. An application modified the network to work as an early warning and real-time marine outfall monitoring system. It was important to configure the network’s scheduled parameters correctly so the network would behave as expected. The network’s performance was evaluated by analysing the simulation log results, an example of the applications of the SIL environment.

The study of the packets’ traffic showed that the node’s workflow described in Section 6.4 worked as expected, with zero packet loss in all simulation iterations. It removed packet loss at the expense of generating duplicated packets below 4% in all cases.

One-way delay results agreed with Equation (4), where median values matched propagation time and the time increment related to flush time was mitigated as the number of hops increased. The network’s ability to detect anomalies and send them as high priority was tested. Alert packets reached a delay per hop 100 times lower than the regular ones. The delay time per hop could increase during transmission failures, and the packets were sent again.

This study allowed us to know not only the number of duplicate packets and the delay time of transmissions but also the increase in energy consumption incurred because of it. We could highlight the correlation between nodes with the highest consumption and nodes with the longest one-way delays. This means that overloaded nodes were subject to more duplicate packets and higher power consumption.

Moreover, by studying the one-way delay, we detected a characteristic to be improved. Some isolated warning packets have taken up to 4 h per hop to reach the master node. The ability to detect this type of malfunction in how the network manages the alarm packets shows that the approach of this platform can test if a complete network is able to run in a real scenario.

The SIL technique proposed in this study therefore represents a practical tool for code reuse, making it possible to establish, test, and validate the performance of an application with production-ready code.

Being focused on a real deployment has provided a complementary perspective where we have been able to use the SIL framework to select proper parameters, report undesired network behaviours, anticipate possible solutions, measure network performance, and find out which nodes are more critical. These steps are of capital importance for designing the real deployment affordably.

## Figures and Tables

**Table 1:** Network’s scheduled parameters.

Parameter	Value
Sample period for regular packets (spm *)	1
Temporal resolution for regular packets (spm)	${}^{1}{\;/\;}_{30}$
Propagation time for regular packets (min)	15
Sample period for warning packets (spm)	1
Temporal resolution for warning packets (spm)	1
Propagation time for warning packets (min)	0
Flush time period for nodes in line topology (min)	30
Flush time period for nodes in mesh topology (min)	60
Delay between branches in mesh topology (min)	30
Simulator time slots (ms)	25
Simulated modem	CML Microcircuits FX614
Modulation	2-FSK
Data rate (bps)	1200
Output power (dBm)	33
Transmission state unitary cost (mWh/s)	1.111
Receiving state unitary cost (mWh/s)	0.472
Idle state unitary cost (mWh/s)	0.416
Channel model	[36]
Receiver sensitivity (dBm)	−120
Communication range (m) for BER 1 × 10${}^{-6}$	18.10
STANAG 5066 maximum chunk size (bytes)	100
STANAG 5066 maximum packet size [including overhead] (bytes)	116

* spm stands for number of samples per minute.

**Table 2:** Node’s scheduled parameters.

Address	Relay	Flush Time	TNV	SNV
0.0.1.0				
0.0.1.1	0.0.1.0	15, 45	18.10–18.80	40-32
0.0.1.2	0.0.1.1	0, 30	18.10–18.80	40-32
0.0.1.3	0.0.1.2	45, 15	18.10–18.80	40-32
0.0.1.4	0.0.1.3	30, 0	18.10–18.80	40-32
0.0.1.5	0.0.1.4	15, 45	18.10–18.80	40-32
0.0.1.6	0.0.1.5	0, 30	18.10–18.80	40-32
0.0.1.7	0.0.1.6	45, 15	18.10–18.80	40-32
0.0.1.8	0.0.1.7	30, 0	18.20–18.90	39-31
0.0.1.9	0.0.1.8	15, 45	18.70–19.40	31-23
0.0.1.10	0.0.1.9	0	19.20–19.90	23-15
0.0.1.11	0.0.1.9	30	19.20–19.90	23-15
0.0.1.12	0.0.1.10	45	19.30–20.00	22-14
0.0.1.13	0.0.1.11	15	19.30–20.00	22-14
0.0.1.14	0.0.1.12	30	19.30–20.00	22-14
0.0.1.15	0.0.1.13	0	19.30–20.00	22-14
0.0.1.16	0.0.1.14	15	19.30–20.00	22-14
0.0.1.17	0.0.1.15	45	19.30–20.00	22-14
0.0.1.18	0.0.1.16	0	19.30–20.00	22-14
0.0.1.19	0.0.1.17	30	19.30–20.00	22-14
0.0.1.20	0.0.1.18	45	19.30–20.00	22-14
0.0.1.21	0.0.1.19	15	19.30–20.00	22-14
0.0.1.22	0.0.1.20	30	19.30–20.00	22-14
0.0.1.23	0.0.1.21	0	19.30–20.00	22-14
0.0.1.24	0.0.1.22	15	19.30–20.00	22-14
0.0.1.25	0.0.1.23	45	19.30–20.00	22-14
0.0.1.26	0.0.1.24	0	19.30–20.00	22-14
0.0.1.27	0.0.1.25	30	19.30–20.00	22-14
0.0.1.28	0.0.1.26	45	19.30–20.00	22-14
0.0.1.29	0.0.1.27	15	19.30–20.00	22-14
0.0.1.30	0.0.1.28	30	19.30–20.00	22-14
0.0.1.31	0.0.1.29	0	19.30–20.00	22-14
0.0.1.32	0.0.1.30	15	19.30–20.00	22-14
0.0.1.33	0.0.1.31	45	19.30–20.00	22-14

## Data Availability

Data of this research is available upon request via corresponding author.

## References

[1] Akyildiz I.F., Pompili D., Melodia T. (2005). Underwater acoustic sensor networks: Research challenges. Ad Hoc Netw..

[2] Heidemann J., Stojanovic M., Zorzi M. (2012). Underwater sensor networks: Applications, advances and challenges. Philos. Trans. R. Soc. A Math. Phys. Eng. Sci..

[3] Felemban E., Shaikh F.K., Qureshi U.M., Sheikh A.A., Qaisar S.B. (2015). Underwater Sensor Network Applications: A Comprehensive Survey. Int. J. Distrib. Sens. Netw..

[4] Gussen C.M., Diniz P.S., Campos M.L., Martins W.A., Costa F.M., Gois J.N. (2016). A survey of underwater wireless communication technologies. J. Commun. Inf. Sys..

[5] Haque K.F., Kabir K.H., Abdelgawad A. (2020). Advancement of Routing Protocols and Applications of Underwater Wireless Sensor Network (UWSN)—A Survey. J. Sens. Actuator Netw..

[6] Acar G., Adams A. (2006). ACMENet: An underwater acoustic sensor network protocol for real-time environmental monitoring in coastal areas. IEE Proc. Radar Sonar Navig..

[7] Kumar P., Kumar P., Priyadarshini P. Underwater acoustic sensor network for early warning generation. Proceedings of the 2012 Oceans.

[8] Kong J., Cui J.h., Wu D., Gerla M. Building underwater ad-hoc networks and sensor networks for large scale real-time aquatic applications. Proceedings of the MILCOM 2005-2005 IEEE Military Communications Conference.

[9] Ahmed M., Salleh M., Channa M.I. (2018). CBE2R: Clustered-based energy efficient routing protocol for underwater wireless sensor network. Int. J. Electron..

[10] Alonso-Eugenio V., Guerra V., Zazo S., Perez-Alvarez I. (2020). Software-in-Loop Simulation Environment for Electromagnetic Underwater Wireless Sensor Networks over STANAG 5066 Protocol. Electronics.

[11] Aguirre J.C., Sarasate J.V., Correa M.D.A. (2007). Reparación del emisario de Las Palmas de Gran Canaria. Rev. Digit. Del Cedex.

[12] Quintana-Díaz G., Mena-Rodríguez P., Pérez-Álvarez I., Jiménez E., Dorta-Naranjo B.P., Zazo S., Pérez M., Quevedo E., Cardona L., Hernández J.J. (2017). Underwater electromagnetic sensor networks—Part I: Link characterization. Sensors.

[13] Zazo J., Macua S., Zazo S., Pérez M., Pérez-Álvarez I., Jiménez E., Cardona L., Brito J., Quevedo E. (2016). Underwater Electromagnetic Sensor Networks, Part II: Localization and Network Simulations. Sensors.

[14] Washburn L., Jones B.H., Bratkovich A., Dickey T., Chen M.S. (1992). Mixing, dispersion, and resuspension in vicinity of ocean wastewater plume. J. Hydraul. Eng..

[15] Petrenko A., Jones B., Dickey T. (1998). Shape and initial dilution of Sand Island, Hawaii sewage plume. J. Hydraul. Eng..

[16] Jones B.H., Barnett A., Robertson G. Towed mapping of the effluent plume from a coastal ocean outfall. Proceedings of the MTS/IEEE Oceans 2001. An Ocean Odyssey. Conference Proceedings (IEEE Cat. No. 01CH37295).

[17] Hunt C., Steinhauer W., Mansfield A., Albro C., Roberts P., Geyer W., Mickelson M. (2002). Massachusetts Water Resources Authority Effluent Outfall Dilution: April 2001. Boston Mass. Water Resour. Authority. Rep. ENQUAD.

[18] Ramos P., Neves M., Pereira F. (2007). Mapping and initial dilution estimation of an ocean outfall plume using an autonomous underwater vehicle. Cont. Shelf Res..

[19] Ramos P., Cunha S., Neves M., Pereira F., Quintaneiro I. (2005). Sewage outfall plume dispersion observations with an autonomous underwater vehicle. Water Sci. Technol..

[20] Bonin-Font F., Campos M.M., Carrasco P.L.N., Codina G.O., Font E.G., Fidalgo E.G. Towards a new methodology to evaluate the environmental impact of a marine outfall using a lightweight AUV. Proceedings of the OCEANS 2017-Aberdeen.

[21] Bonin-Font F., Lalucat J., Oliver-Codina G., Massot-Campos M., Font E.G., Carrasco P.L.N. (2018). Evaluating the impact of sewage discharges on the marine environment with a lightweight AUV. Mar. Pollut. Bull..

[22] Adamo F., Attivissimo F., Carducci C.G.C., Lanzolla A.M.L. (2014). A smart sensor network for sea water quality monitoring. IEEE Sens. J..

[23] King P., Venkatesan R., Cheng L. A study of channel capacity for a seabed underwater acoustic sensor network. Proceedings of the OCEANS.

[24] Person R., Blandin J., Stout J., Briole P., Ballu V., Etiope G., Ferentinos G., Masson M., Smolders S., Lykousis V. ASSEM: A new concept of observatory applied to long term SEabed Monitoring of geohazards. Proceedings of the Oceans 2003. Celebrating the Past... Teaming Toward the Future (IEEE Cat. No.03CH37492).

[25] Chandrasekhar V., Seah W.K., Choo Y.S., Ee H.V. Localization in underwater sensor networks: Survey and challenges. Proceedings of the 1st ACM International Workshop on Underwater Networks.

[26] Tan H.P., Eu Z.A., Seah W.K. An enhanced underwater positioning system to support deepwater installations. Proceedings of the OCEANS.

[27] Akyildiz I.F., Wang P., Sun Z. (2015). Realizing underwater communication through magnetic induction. IEEE Commun. Mag..

[28] Che X., Wells I., Dickers G., Kear P., Gong X. (2010). Re-evaluation of RF electromagnetic communication in underwater sensor networks. IEEE Commun. Mag..

[29] Domingo M.C. (2012). Magnetic induction for underwater wireless communication networks. IEEE Trans. Antennas Propag..

[30] Namenas A., Kaak T., Schmidt G. (2017). Real-time simulation of underwater acoustic channels. Fortschritte der Akustik–DAGA.

[31] Das A.P., Thampi S.M. (2016). Simulation tools for underwater sensor networks: A survey. Netw. Protoc. Algorithms.

[32] Egea-Lopez E., Vales-Alonso J., Martinez-Sala A.S., Pavon-Marino P., García-Haro J. (2005). Simulation tools for wireless sensor networks. Summer Simulation Multiconference, SPECTS.

[33] The Network Simulator, NS–2. http://www.isi.edu/nsnam/ns/.

[34] Xie P., Zhou Z., Peng Z., Yan H., Hu T., Cui J.H., Shi Z., Fei Y., Zhou S. Aqua-Sim: An NS-2 based simulator for underwater sensor networks. Proceedings of the OCEANS.

[35] Martin R., Rajasekaran S., Peng Z. Aqua-Sim Next generation: An NS-3 based underwater sensor network simulator. Proceedings of the International Conference on Underwater Networks & Systems.

[36] Baldo N., Maguolo F., Miozzo M., Rossi M., Zorzi M. ns2-MIRACLE: A modular framework for multi-technology and cross-layer support in network simulator 2. Proceedings of the 2nd International Conference on PERFORMANCE Evaluation Methodologies and Tools.

[37] Petrioli C., Petroccia R., Shusta J., Freitag L. From underwater simulation to at-sea testing using the ns-2 network simulator. Proceedings of the OCEANS 2011 IEEE-Spain.

[38] Petrioli C., Petroccia R., Potter J.R., Spaccini D. (2015). The SUNSET framework for simulation, emulation and at-sea testing of underwater wireless sensor networks. Ad Hoc Netw..

[39] Masiero R., Azad S., Favaro F., Petrani M., Toso G., Guerra F., Casari P., Zorzi M. DESERT Underwater: An NS-Miracle-based framework to design, simulate, emulate and realize test-beds for underwater network protocols. Proceedings of the 2012 Oceans-Yeosu.

[40] Zuba M., Jiang Z., Yang T., Su Y., Cui J.H. An advanced channel framework for improved underwater acoustic network simulations. Proceedings of the 2013 OCEANS-San Diego.

[41] Peng Z., Zhou Z., Cui J.H., Shi Z.J. Aqua-Net: An underwater sensor network architecture: Design, implementation, and initial testing. Proceedings of the OCEANS.

[42] Le S.N., Peng Z., Cui J.H., Zhou H., Liao J. (2013). SeaLinx: A multi-instance protocol stack architecture for underwater networking. Proceedings of the Eighth ACM International Conference on Underwater Networks and Systems.

[43] Chitre M., Bhatnagar R., Soh W.S. UnetStack: An agent-based software stack and simulator for underwater networks. Proceedings of the 2014 Oceans-St. John’s.

[44] Zhu Y., Pu L., Wang Z., Lu X., Martin R., Luo Y., Peng Z., Cui J.H. Underwater acoustic network protocol stacks: Simulator-based vs. OS-based. Proceedings of the 2014 Oceans-St. John’s.

[45] Dhviya V., Arthi R. (2014). Analysis of simulation tools for underwater wireless sensor networks. Int. J. Comput. Sci. Eng. Technol. (IJCSET).

[46] Raj C., Sukumaran R. (2015). Modeling UWSN simulators–a taxonomy. Int. J. Comput. Inf. Eng..

[47] Monika M., Rangaswamy D.S. (2015). A study on under water network simulators. Int. J. Technol. Enhanc. Emerg. Eng. Res..

[48] Nayyar A., Balas V.E., Bhattacharyya S., Hassanien A.E., Gupta D., Khanna A., Pan I. (2019). Analysis of Simulation Tools for Underwater Sensor Networks (UWSNs). International Conference on Innovative Computing and Communications.

[49] Issac A., Samad S.A., Jereesh A. Software tools for simulation and realization of underwater networks. Proceedings of the 2017 International Conference on Communication and Signal Processing (ICCSP).

[50] Chitre M., Freitag L., Sozer E., Shahabudeen S., Stojanovic M., Potter J. (2006). An Architecture for Underwater Networks.

[51] Shahabudeen S., Chitre M., Motani M., Siah A.L.Y. Unified simulation and implementation software framework for underwater MAC protocol development. Proceedings of the OCEANS 2009.

[52] Demers S., Gopalakrishnan P., Kant L. A generic solution to software-in-the-loop. Proceedings of the MILCOM 2007-IEEE Military Communications Conference.

[53] Emisario submarino El Teatro, Censo de Vertidos Desde Tierra al Mar en Canarias. https://www.idecanarias.es/resources/Vertidos_2017/Fichas/GranCanaria/137.pdf.

[54] DS18B20 Datasheet, Programmable Resolution 1-Wire Digital Thermometer. https://datasheets.maximintegrated.com/en/ds/DS18B20.pdf.

[55] Coutinho R.W., Boukerche A., Vieira L.F., Loureiro A.A. (2018). Underwater wireless sensor networks: A new challenge for topology control–based systems. ACM Comput. Surv. (CSUR).

[56] Cario G., Casavola A., Gjanci P., Lupia M., Petrioli C., Spaccini D. Long lasting underwater wireless sensors network for water quality monitoring in fish farms. Proceedings of the OCEANS 2017-Aberdeen.

[57] Oceanographic and Weather Continuous Time Series of PLOCAN. http://siboy.plocan.eu/buoy.

[58] Millero F.J., Chen C.T., Bradshaw A., Schleicher K. (1980). A new high pressure equation of state for seawater. Deep Sea Res. Part A Oceanogr. Res. Pap..

[59] Almes G., Kalidindi S., Zekauskas M. (1999). A One-Way Delay Metric for IPPM.

